# The Prevalence and Assessment of ErbB2-Positive Breast Cancer in Asia: A Literature Survey

**DOI:** 10.1002/cncr.25476

**Published:** 2010-08-16

**Authors:** Yew Oo Tan, Sehwan Han, Yen-Shen Lu, Cheng-Har Yip, Patrapim Sunpaweravong, Joon Jeong, Priscilla B Caguioa, Shyam Aggarwal, Ee Min Yeoh, Hanlim Moon

**Affiliations:** 1 Medical Oncology Center, Gleneagles Medical CenterSingapore; 2 Breast Cancer Center, Inje University Sanggye Paik HospitalSeoul, Korea; 3 Department of Oncology, National Taiwan University HospitalTaipei, Taiwan; 4 Department of Surgery, University Malaya Medical CentreKuala Lumpur, Malaysia; 5 Medical Oncology Unit, Songklanagarind HospitalHat Yai, Songkhla, Thailand; 6 Department of Surgery, Gangnam Severance Hospital, Yonsei UniversitySeoul, Korea; 7 Cancer Institute, St.Luke's Medical CenterQuezon City, The Philippines; 8 Department of Oncology, Sir Ganga Ram HospitalNew Delhi, India; 9 Research and Development, Oncology, GlaxoSmithKline Asia PacificSingapore

**Keywords:** Asia, breast neoplasms, immunohistochemistry, fluorescence in situ hybridization, erbB-2 receptor

## Abstract

Overexpression of the epidermal growth factor receptor-related gene *ErbB2* occurs in 18% to 25% of patients with breast cancer in Western countries and is associated with a poor prognosis. The prevalence of ErbB2-positive tumors in Asia is unclear, partly because data are limited. The objective of this review was to summarize the reported prevalence of ErbB2-positive tumors from a large sample of Asian patients and to examine ErbB2 assessment methods in Asia. From searches of MEDLINE, local language journals, and local and international conference proceedings as well as locoregional breast cancer experts' recommendations, the authors selected up to 5 studies each from India, Korea, Malaysia, the Philippines, Singapore, Taiwan, and Thailand that reported ErbB2 results based on assessment with immunohistochemistry (IHC) and/or fluorescence in situ hybridization (FISH). The reported prevalence of ErbB2-positive tumors in 22 studies on 24,671 patients, of whom 14,398 patients were assessed for ErbB2 status, varied widely (range, 6%-65%) as did the assessment methods used. Most studies (n = 21) used IHC to assess ErbB2 status, but definitions for positivity varied. When robust assessment methods were used, the median prevalence was 19% based on strong IHC staining (IHC3+; n = 9812 patients) and 25% based on FISH (n = 681 patients). Data on the prevalence of ErbB2-positive breast cancer in Asia are limited. The current survey indicated that the prevalence in Asia may be similar to that in Western countries; thus, up to 1 in 4 Asian patients with breast cancer potentially could benefit from ErbB2-targeted treatment. A standard, reliable ErbB2 assessment method available to patients across Asia is urgently required. ***Cancer* 2010;116:5348–57**. © *2010 American Cancer Society*.

Although the incidence of breast cancer in some Western countries has fallen recently, its incidence still is increasing in Asia.^1^,^2^ Furthermore, the survival rate among patients with breast cancer in Asia is approximately half that of patients in Western countries.^2^,^3^ These observations are driving interest in gaining a better understanding of breast cancer in Asia.

Studies from Western countries indicate that breast cancer survival is low in patients with tumors that over express the human epidermal growth factor receptor-related gene *ErbB2* (*c-ErbB2*, *HER2*, or *neu*).^4^,^5^ Tumors that over express *ErbB2* are more likely to recur^4^,^5^ and are relatively resistant to many treatments.^4^ Two recently developed treatments that specifically target ErbB2, trastuzumab and lapatinib, are effective either alone or with chemotherapy in reducing tumor recurrence and mortality in patients with ErbB2-positive breast cancer.^6^

Given the increasing incidence of breast cancer in Asia and the clinical consequences of ErbB2-positive breast cancer, insight into the prevalence of ErbB2-positive tumors in Asia is important. Recently, 2 large studies of breast cancer registry data in the United States reported that women of Asian descent were more likely to have ErbB2-positive tumors than Caucasian women,^7^,^8^ suggesting possible racial differences. Whether the prevalence of ErbB2-positive breast cancer differs between Asian and Western countries requires clarification. A higher prevalence of ErbB2-positivity may be 1 of several factors contributing to the lower survival rate of Asian breast cancer patients.

Because ErbB2 status is assessed by different methods (by immunohistochemistry [IHC] or, less commonly, by fluorescence in situ hybridization [FISH]^9^), it is also important to know how ErbB2 is assessed within different Asian countries. The accurate estimation of the prevalence of ErbB2-positive tumors in Asia has been hindered by variability in both the availability of assessment methods and the definition of ErbB2 positivity. In addition, data on ErbB2 positivity in Asia are limited in the literature that can be searched electronically (eg, in the MEDLINE database).

To gain insight into the prevalence and assessment of ErbB2-positive breast cancer in Asia through a literature survey, we formed the Early Breast Cancer Working Group (EBCWG). Because we anticipated that it would be a challenge to find data on ErbB2 prevalence in Asia, we also engaged with our peers who were familiar with relevant research in our countries. The objectives of our literature survey were to summarize the reported prevalence of ErbB2-positive breast cancer in 7 Asian countries and to examine the ErbB2 assessment methods used in these countries.

## MATERIALS AND METHODS

### Literature Search Strategy

After the initial EBCWG meeting, we conducted separate literature searches for our respective countries (India, Korea, Malaysia, the Philippines, Singapore, Taiwan, and Thailand). We obtained relevant literature from searches of the MEDLINE database using PubMed (2000 to September 2008) with the search terms (ErbB2 OR HER2 OR ErbB-2 OR HER-2) AND “breast cancer” AND (country of interest). We also searched, either electronically or by hand, local language journals and the proceedings of local and international oncology conferences, and we consulted with our professional networks for access to results from recent studies. Because the availability and quality of ErbB2 assessment in Asian clinics increased after the approval of trastuzumab in 2000, we limited our searches to studies that were published during or after 2000.

### Study Selection Criteria

We selected studies that reported the results of ErbB2 assessment by IHC, FISH, or both for further review. To focus on clinical ErbB2 assessment in our countries, we excluded studies of Asian patients who were living in Western countries and studies that reported serum, messenger RNA, or cell line ErbB2 data. For each country, we selected up to 5 studies for detailed review. Preference was given to studies that had large sample sizes, multicenter studies, and studies that were published in higher impact factor journals.

### Data Analysis

The EBCWG met again to analyze data from the selected studies. We divided the ErbB2 prevalence data from the studies into 3 categories based on the testing and grading methods used for assessment: 1) IHC combined or not specified, ie, IHC with intermediate (IHC2+) and strong (IHC3+) staining combined or IHC with no grading method specified; 2) IHC3+ only; and 3) FISH. Then, we calculated the median and mean (±standard deviation) prevalence of ErbB2-positive tumors in each category.

We hypothesized that lower income countries may report higher ErbB2+ prevalence rates because of the limited availability of robust, but expensive, assay methods (eg, FISH). We used a 2-sample *t* test (assuming unequal variances) to compare differences between low/middle-income countries and high-income countries in the reported ErbB2+ prevalence rates assessed with IHC2+/IHC3+ combined or not specified, IHC3+ only, or FISH. Countries were classified as low, middle, or high income based on the 2007 World Bank income classification (available at: http://data.worldbank.org/about/country-classifications/a-short-history accessed July 23, 2010).

## RESULTS

### Summary of Selected Literature

We selected 22 studies (see Table 1), including 17 publications^10-26^ and 5 conference abstracts,^27-31^ for further review. For Malaysia^30^,^31^ and the Philippines,^29^ only conference abstracts met our selection criteria.

**Table 1:** Description of 22 Studies Reporting Human Epidermal Growth Factor Receptor-Related Gene 2-Positive Breast Cancer in Asia

	Sampling			Patients (%)	
Study and Design	No.	Setting	Dates	Age	Stage or Grade^a^
**India**					
Bhamrah 2008^27^; observational	210	All India Institute of Medical Sciences	NR	NR	NR
Meenakshi 2003^17^; prospective, observational	127	Cancer Institute	NR	NR	NR
**Korea**					
Kim 2006^14^; retrospective, observational	776	Asan Medical Center	1993-1998	Mean, 47.4 y; range, 24-88 y	Stage: I, 182 (23.5); II, 336 (47.2); III, 225 (29); IV, 3 (0.4)
Korean Breast Cancer Society, 2006^24^; retrospective, observational	Total, 9668; ErbB2- tested, 4318	Medical schools, 39; hospitals, 27; private clinics, 7	2004	Age known: 8376; median, 47 y; ≤29 y, 157 (1.9); 30-39 y, 1409 (16.8); 40-49 y, 3452 (41.2); 50-59 y, 1987 (23.7); 60-69 y, 1017 (12.1); ≥70 y, 354 (4.2)	Total assessed, 5301 (100); Stage: 0, 508 (9.6); I, 1889 (35.6); II, 2062 (38.9); III, 772 (14.6); IV, 70 (1.3)
Park 2003^19^; prospective, observational	188	University Sanggye Paik Hospital	NR	NR	Histologic grade^b^: 1, 22 (11.7); 2. 83 (44.1); 3. 73 (38.8); unknown: 10 (5.3)
Park & Han 2004^18^; observational	Samples in TMA, 132-261	Inje University Sanggye Paik Hospital	NR	NR	NR
Shin 2006^22^; retrospective, observational	Total, 4063; ErbB2- tested, 2196	Seoul National Universiti Hospital	1981-2002	Median, 46 y; ≤29 y, 163 (4); 30-39 y, 983 (24.2); 40-49 y, 1572 (38.7); 50-59 y, 921 (22.7); 60-69 y, 334 (8.2); ≥70 y, 79 (1.9); unknown, 16 (0.4)^c^	Total assessed, 3761 (100); Stage: 0, 175 (4.7); I, 1042 (27.7); II, 1652 (43.9); III, 726 (19.3); IV, 166 (4.4)
**Malaysia**					
Shahrun 2008^30^; retrospective, observational	300	International Islamic University Malaysia; Universiti Kebangsaan Malaysia	2003-2007	NR	NR
Tan &Yip 2008^31^; prospective, observational	393	University Malaya Medical Center	2007	<40 y (12); ≥40 y (88)	Stage: 0, 11 (2.7); I, 98 (24.8); II, 189 (48.2); III, 61 (15.5); IV, 34 (8.7)
**Singapore**					
Fernandopulle 2006^12^; retrospective, observational	112	Singapore General Hospital	1993-2004	All, ≤35 y; mean, 29.1 y; median, 30 y; range, 19-35 y	Histologic grade^d^: 1, 7 (6.3); 2, 24 (21.4); 3, 54 (48.2); unknown, 27 (24.1)
Selvarajan 2006^20^; retrospective, observational	184	Singapore General Hospital	1998-2002	NR	NR
Selvarajan 2006^21^; retrospective, observational	321	Singapore General Hospital	1998-2002	≤50 y; 134 (41.7); >50 y, 181 (56.4); unknown, 6 (1.9)	Stage: I, 68 (21.2); II, 102 (61.8); III, 23 (13.9); IV, 5 (3)
Tan 2008^23^; retrospective, observational	165	National Cancer Centre Tissue Repository	2000-2004	Mean±SD, 56±12 y; range, 30-79 y; ≤54 y, 82 (49.7); >54 y, 83 (50.3)	Stage: I, 36 (21.8); II, 192 (59.8); III, 28 (8.7); IV, 7 (2.2); unknown, 26 (8.1)^e^
Zhang 2003^26^; retrospective, observational	97	National University Hospital	NR	NR	Histologic grade^f^: 1, 9 (9.3); 2, 34 (35.1); 3, 44 (45.4); unknown, 10 (10.3)
**Taiwan**					
Chen 2008^11^; open label, phase 2 trial	63	Chang Gung Memorial Hospital	2002-2005	Median, 46 y; range, 29-69 y	Tumor classification: T2, 25 (39.7); T3, 32 (50.8); T4, 6 (9.5)
Huang 2008^13^; prospective pilot trial	192	Kaohsiung Medical University Hospital	2003-2005	Median±SD, 48.9±9.6 y; <50 y, 112 (58.3); ≥50 y, 80 (41.7)	Stage: II, 160 (83.3); III, 32 (16.7)
Lin 2007^16^; Phase 2 randomized controlled trial	101	Chang Gung Memorial Hospital	2000-2002	Median, 48 y; range, 25-70 y	All stage IV
Lin 2009^15^; prospective, observational	1028	National Taiwan University Hospital	2004-2006	Median, 50 y; range, 23-88 y	Stage: I, 316 (30.7); II, 417 (45.8); III, 176 (17.1); IV, 52 (5.1); unknown, 13 (1.3)
Tsai 2001^25^; retrospective, observational	167	Kaohsiung Medical University Hospital	1990-1999	Mean age: Familial, 47.8 y, n=56; nonfamilial, 50.1 y, n=111	Histologic grade^g^: 1, 13 (7.8); 2, 51 (30.5); 3, 63 (37.7); unknown, 40 (24)
**Thailand**					
Chearskul 2001^10^; retrospective, observational	506	Siriraj Hospital	1992-2000	Mean age, 50.8 y; range, 24-89 y; ≤50 y, 246 (48.6); >50 y, 255 (50.4); unknown, 5 (1)	I, 50 (9.9); II, 366 (72.3); III, 60 (11.9); IV, 5 (1); unknown, 25 (4.9)
Moohamad 2008^28^; retrospective, observational	442	Phramongkutklao Hospital	2003-2006	NR	NR
**The Philippines**					
Sanchez 2007^29^; retrospective, observational	Total, 5307; ErbB2- tested, 2333	St. Luke's Medical Center	1994-2004	NR	NR

NR indicates not reported; ErbB2, human epidermal growth factor receptor-related gene 2; TMA, tissue microarray; SD, standard deviation.

a American Joint Committee on Cancer stages unless otherwise noted.

b Nottingham histologic grading system.

c Percentages listed were calculated on a total of 4063 patients, as reported by the article. Note: number of patients listed by age in the article actually totals 4068.

d United Kingdom National Health System grading system.

e The number of patients listed by tumor stage in the article totals 166, not 165. Percentages listed here were calculated based on a total of 165 patients.

f Tumor grades were stated in the article, but the grading system was not specified.

g Modified Bloom-Richardson grading system.

### Patients Assessed for ErbB2

Our selected studies included 24,671 patients, including 14,398 patients (58.4%) who were assessed for ErbB2 (Table 1). The number of patients in each study ranged from 63 patients^11^ to 9668 patients,^24^ and the number assessed for ErbB2 ranged from 63 patients^11^ to 4319 patients.^24^ The age of patients varied widely (range, 19-89 years) among those studies in which it was reported; however, most patients were in their 40s or 50s. Most patients (10,203 of 13,171 patients; 77.5%) for whom cancer stage was reported had early stage breast cancer (stage 0, n = 694 [5.3%]; stage I, n = 3732 [28.3%]; stage II, n = 5777 [43.9%]).

### Prevalence of ErbB2-Positive Breast Cancer

**Figure 1:**
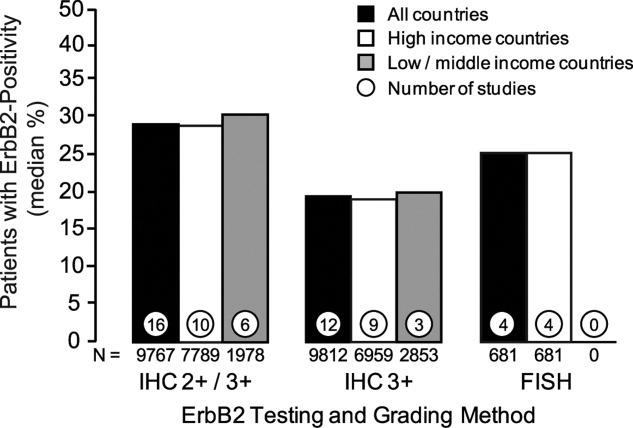
The median prevalence of human epidermal growth factor receptor-related gene 2 (ErbB2)-positive breast cancer is shown. Solid columns indicate 7 Asian countries; open columns, high-income countries; hatched columns, low-income/middle-income countries. The number of studies and the number of patients are shown within and beneath each column, respectively. IHC indicates immunohistochemistry; FISH, fluorescence in situ hybridization.

The prevalence of ErbB2-positive breast cancer in Asia was influenced by the testing method and grading criteria used (Tables 2 and 3). Generally, prevalence results were lowest with IHC when positivity was defined as IHC3+, intermediate with FISH, and highest with IHC when positivity was defined as IHC2+ and IHC3+ combined or when positivity was not defined. When the results obtained using any of the assessment methods were examined, the prevalence of ErbB2-positive breast cancer varied widely (range, 6%-65%). When results were limited to those obtained with the more robust methods (IHC3+, FISH), the median prevalence was 19% and 25%, respectively (Fig. 1). Even with this narrower focus, these results were obtained from 10,076 patients in 5 Asian countries (India, Korea, Singapore, Taiwan, and the Philippines). The least variable results were obtained with the less common assessment method, FISH (Table 3).

**Table 2:** Description of Human Epidermal Growth Factor Receptor-Related Gene 2 (ErbB2) Assessment in 22 Studies Reporting ErbB2-Positive Breast Cancer in Asia

		Percentage of Patients (No./Total No.)		
Study	ErbB2 Assessment	IHC2+ and IHC3+^a^	IHC3+^b^	FISH
**India**				
Bhamrah 2008^27^	IHC: antibody, NR	28.6 (60/210)^c^,^d^	NR	NR
Meenakshi 2003^17^	IHC2+, IHC3+: antibody, in-house monoclonal (CIBCgp185)	25.2 (32/127)	15.7 (20/127)	NR
**Korea**				
Kim 2006^14^	IHC3+: ≥10% cells positive; antibody, Dako (Glostrup, Denmark)	NR	25 (194/776)	NR
Korean Breast Cancer Society 2006^24^	IHC2+, IHC3+; antibody: NR	36.9 (1594/4319)	19.8 (854/4319)	NR
Park 2003^19^	FISH: signal ≥2-fold centromere region of chromosome 17; CISH: gene copy >4	NR	NR	24.5 (46/188)
Park & Han, 2004^18^	IHC2+, IHC3+: antibodies, HercepTest (Dako), rabbit anti-c-ErbB2 (Zymed, South San Francisco, Calif), mouse anti-c-ErbB2 (Zymed); FISH: criteria not reported in English	17.9-22.7 (40/188)^e^	6.1-14.4 (21/188)^e^	24-28 (73/261)^e^,^f^
Shin 2006^22^	IHC2+, IHC3+: antibody, NR	51.5 (1131/2196)	NR	NR
**Malaysia**				
Shahrun 2008^30^	NR	44.4 (133/300)^c^,^g^	NR	NR
Tan & Yip, 2008^31^	IHC2+, IHC3+: antibody, Dako	60.6 (238/393)^c^	34.5 (136/393)^c^	NR
**Singapore**				
Fernandopulle 2006^12^	IHC2+, IHC3+: antibody, rabbit polyclonal (A0485; Dako)	29.6 (16/54)	NR	NR
Selvarajan 2006^20^	IHC2+, IHC3+: antibody, rabbit polyclonal, Dako (A0485; Dako); performed on standard sections and TMA	21.2 (39/184)	12.5 (23/184)^h^	NR
Selvarajan 2006^21^	IHC2+, IHC3+: antibody, rabbit polyclonal (Dako)	34.3 (110/321)	NR	NR
Tan 2008^23^	IHC2+, IHC3+: antibody: SP3, Lab Vision (Thermo Fisher Scientific, Waltham, Mass); FISH: signal ≥2.2-fold centromere region of chromosome 17 (PathVysion, Des Plaines, Ill)	19 (27/142)	11.3 (16/142)	37.9 (55/145)
Zhang 2003^26^	IHC2+, IHC3+: antibody, HercepTest; FISH: signal ≥2-fold centromere region of chromosome 17 (PathVysion)	25 (23/92)	15.2 (14/92)	23 (20/87)
**Taiwan**				
Chen 2008^11^	IHC3+: antibody, HercepTest	NR	19 (12/63)	NR
Huang 2008^13^	NR	64.6 (124/192)^g^	NR	NR
Lin 2007^16^	NR	27.7 (28/101)^g^	NR	NR
Lin 2009^15^	IHC3+: antibody, polyclonal (Dako); IHC2+: confirmation by FISH (signal ≥2-fold centromere region of chromosome 17; PathVysion)	NR	20.5 (211/1028)^i^	NR
Tsai 2001^25^	IHC3+: antibody, HercepTest	NR	41.3 (69/167)	NR
**Thailand**				
Chearskul 2001^10^	IHC2+: antibody, rabbit polyclonal (Dako)	32.2 (163/506)	NR	NR
Moohamad 2008^28^	IHC: antibody, NR	17.9 (79/442)^g^	NR	NR
**The Philippines**				
Sanchez 2007^29^	IHC3+: antibody, NR	NR	20 (466/2333)	NR

IHC indicates immunohistochemistry; FISH, fluorescence in situ hybridization; CISH, chromogenic in situ hybridization; TMA, tissue microarray; NR, not reported.

a Combined results of IHC2+ and IHC3+ were reported (whether separately or together) unless indicated otherwise.

b Results for IHC3+ were reported separately.

c The number of ErbB2-positive patients was not stated in study but was calculated from the number assessed and prevalence.

d Results are reported as a range.

e This study used 3 antibodies; results using the HercepTest are shown.

f This study used FISH in 3 separate TMAs; results from the largest sample (N=261) are shown.

g IHC results were reported as positive, but criteria were not specified.

h Results for standard sections.

i Included IHC2+ confirmed by FISH (50 of 211).

**Table 3:** Prevalence of Human Epidermal Growth Factor Receptor-Related Gene 2-Positive Breast Cancer in Asia

	ErbB2 Assessment Method		
Characteristic	IHC2+ and 3+^a^	IHC3+^b^	FISH
Studies using method, no.^c^	16	12	4
ErbB2-positive patients, no./total no.^c^	3837/9767	2036/9812	194/681
**ErbB2-positive prevalence, %**			
Minimum	17.9	6.1	23
Maximum	64.6	41.3	37.9
Median	29.1	19.4	25.3
Mean±SD	33.7±14.5	20.4±9.3	27.9±6.8

ErbB2 indicates human epidermal growth factor receptor-related gene 2; IHC, immunohistochemistry; FISH, fluorescence in situ hybridization; SD, standard deviation.

a Either combined IHC2+ and IHC3+ (whether reported separately or together) or IHC results were reported as positive, but criteria were not specified.

b Results for IHC3+ were reported separately.

c Because some studies used more than 1 ErbB2 assessment method, the apparent total number of studies and patients (ie, the sum of IHC2+/IHC3+, IHC 3+, and FISH shown in this table) is greater than the actual number.

The mean prevalence of ErbB2-positive tumors was similar to the median prevalence for all 3 assessment methods (Table 3). Omitting the maximum and minimum prevalence values did not change the mean markedly (IHC2+/IHC3+ combined or not specified = 32.6% vs an actual rate of 33.7%; IHC3+ only = 19.4% vs an actual rate of 20.4%).

When prevalence data from high-income (Korea, Singapore, and Taiwan) and low or middle-income (India, Malaysia, Thailand, and the Philippines) countries were compared, there was no significant difference (*P* = .82) in the mean prevalence when positivity was defined as IHC2+/IHC3+ or when the definition was not specified. Similarly, there was no significant difference (*P* = .59) in the mean prevalence when positivity was defined as IHC3+. The similarity in prevalence results between high-income and low or middle-income countries also was apparent from a comparison of the median values for IHC (Fig. 1). No comparison could be made for studies using FISH, because none of the studies from low or middle-income countries used this method. Among the countries in our survey, only high-income countries (ie, Korea, Singapore, and Taiwan) routinely tested for ErbB2 status and subsidized the cost of trastuzumab for eligible patients (Table 4).

**4:** Availability of Human Epidermal Growth Factor Receptor-Related Gene 2 (ErbB2) Assessment Methods and ErbB2-Targeted Treatments in 7 Asian Countries

	High-Income Countries			Low/Middle-Income Countries			
Variable	Korea	Singapore	Taiwan	India	Malaysia	Philippines	Thailand
**IHC testing**							
Routine?	Yes	Yes	Yes	Yes	No	No	No
Standardized?	Yes	Yes	No	No	No	No	Partial
**FISH testing**							
Routine?	Yes	Yes	Yes	No	No	No	No
Standardized?	Partial	Partial	Partial	No	No	No	Partial
**ErbB2-targeted treatment**							
Subsidized?	Yes	Yes	Yes	Partial	Partial	No	Partial

IHC indicates immunohistochemistry; FISH, fluorescence in situ hybridization.

### ErbB2 Assessment Methods

The most common ErbB2 assessment method was IHC, which was used in 21 of 22 studies (95.5%). Although 12 studies provided the name and/or supplier of the antibody used, 7 studies (including the 5 conference abstracts) did not specify either. The most commonly used antibodies were from Dako (Glostrup, Denmark), including 4 studies that used the HercepTest (Dako), 2 studies that used the A0485 rabbit polyclonal antibody, and 4 studies that used unspecified Dako antibodies. Only 4 studies^18^,^19^,^26^,^31^ from 2 countries (Korea and Singapore) used FISH to assess ErbB2, whereas 1 study from Taiwan^15^ used FISH to confirm ErbB2 positivity in tumors with IHC2+ staining.

## DISCUSSION

On the basis of the ErbB2 assessment results from 14,398 patients in 7 Asian countries, our literature survey indicated that the reported prevalence of ErbB2-positive breast cancer has varied from 6% to 65%. However, when ErbB2 was assessed robustly using IHC3+ or FISH, the median prevalence, based on the results from 10,076 Asian patients in 5 countries, was 19% with IHC3+ and 25% with FISH. These prevalence results are similar to those in Western countries.^4^,^5^ Because ErbB2 status is predictive for both prognosis and therapeutic response, knowledge of a patient's ErbB2 status could help physicians identify the most appropriate treatment. Our survey indicates that up to 1 in 4 Asian patients with breast cancer potentially could benefit from ErbB2-targeted treatment. In addition, the variability in ErbB2 assessment methods evident from our survey highlights the need for Asian countries to use a standard, reliable method to assess ErbB2. Enhanced ErbB2 assessment methods would enable us to further refine our understanding of the prevalence of ErbB2-positive breast cancer in Asia.

To the best of our knowledge, this is the first survey of ErbB2-positivity results obtained from a large sample of patients to indicate that the prevalence of ErbB2-positive breast cancer in Asia is similar to that in Western countries (range, 18%-25%).^4^,^5^ Notably, this finding is based on Asian data that were obtained using the more robust assessment methods, IHC3+ and FISH.^32^,^33^ Because the prevalence of ErbB2-positive tumors is not apparently higher in Asia, alternative reasons for the low breast cancer survival rate should be explored. Possible reasons include advanced stage at diagnosis^3^,^34^; younger patients (aged <50 years; associated with more aggressive tumors)^3^,^35^; higher prevalence of estrogen or progesterone receptor-negative tumors, which are less responsive to endocrine therapies^3^,^36^; and the cost and availability of treatments, including those that target ErbB2.^3^,^34^

In our survey, we observed considerable variability in ErbB2 assessment methods. The most common method was IHC; however, many factors contribute to variability in IHC results, including the antibody used^4^,^9^ and the grading criteria.^9^ The relatively small size of many Asian laboratories also may have contributed to the variability we observed. Indeed, the concordance in IHC results between small, local laboratories and high-throughput, central laboratories can be unacceptably low.^9^

We also observed great variability in how IHC results were reported. Many studies reported both IHC2+ and IHC3+ staining separately. However, although some categorized both IHC2+ and IHC3+ staining as positive,^12^,^17^,^18^,^20^,^21^ others categorized only IHC3+ staining as positive.^11^,^14^,^25^,^29^ Recent guidelines that were published jointly by the American Society for Clinical Oncology and the College of American Pathologists (ASCO/CAP) recommend reporting IHC2+ and IHC3+ staining separately and confirming the ErbB2 status of IHC2+ tumors by FISH.^9^ These recommendations are based on the poor concordance between IHC2+ staining and gene amplification by FISH.^4^,^9^,^32^,^33^ Among the studies in our survey, only 5 used FISH to determine or confirm ErbB2 status.^15^,^18^,^19^,^26^,^31^ Not surprisingly, these studies were conducted in Korea, Singapore, and Taiwan, countries that are able to fund this more expensive method.

Despite the apparent similarity in the prevalence of ErbB2-positive breast cancer in Asian and Western countries, many Asian patients will not have the same opportunity as Western patients to receive ErbB2-targeted treatment. ErbB2-positive tumors respond well to ErbB2-targeted treatments,^6^ but these treatments are expensive and, thus, may not be available to many Asian patients (Table 4). Without subsidies, many Asian patients with ErbB2-positive breast cancer may not be able to afford ErbB2-targeted treatments, which in turn, may reduce the incentive for ErbB2 assessment. Individual countries need to weigh the costs of ErbB2 assessment and treatment with the potential benefit for patients. Because our survey suggests that the prevalence of ErbB2-positive tumors is not related to country income level, at least there is no epidemiologic justification for restricting ErbB2 assessment and treatment in lower income countries.

Worldwide standardization of ErbB2 assessment is an important goal not only for obtaining unbiased data on the prevalence of ErbB2-positive breast cancer but also to ensure that patients receive the most appropriate treatment. Ideally, a standardized assessment method would adhere to the ASCO/CAP guidelines.^9^ However, we must emphasize that the stringent ASCO/CAP recommendations are not practical in many lower income countries.^9^ We believe that greater standardization of ErbB2 assessment is particularly needed in the low or middle-income countries that were included in our study (India, Malaysia, Thailand, and the Philippines)(Table 4). The Breast Health Global Initiative recognizes that the costs of ErbB2 assessment, especially FISH, and ErbB2-targeted therapies are “prohibitively expensive” in countries with limited resources.^37^ Because ErbB2 assessment is not routine in many Asian countries, it would be beneficial, if not prudent, to provide pathologists with a standardized protocol and regular validation testing. Given the impact of pathologist and laboratory experience on ErbB2 results, smaller Asian countries also might consider establishing a central laboratory for national ErbB2 assessment.

Our survey has strengths and limitations. We believe that our conclusions regarding the prevalence of ErbB2-positive breast cancer and ErbB2 assessment methods in Asia are strengthened by the use of data from 22 studies involving more than 14,000 patients in 7 Asian countries that vary in ethnicity, size, and economic status. In addition, as experts in our respective countries, we were able to include data from local language journals and conferences, which are not readily available from the MEDLINE database. However, we recognize that our survey does have limitations. We did not perform a comprehensive, systematic literature review; rather, we chose to limit the number of studies we included from each country to avoid over-representation of larger, wealthier countries with more available data. For some countries in our survey, the only available studies that met our criteria were published as conference abstracts. We chose to include these studies, despite their preliminary nature, to examine the current state of ErbB2 assessment and prevalence in these countries. Although data for Malaysia were limited at the time of our survey, the prevalence of ErbB2 positivity reported in a recent article is consistent with the prevalence we obtained.^38^ Also, although our survey included data from 7 countries, we did not include data from all Asian countries. Notably, we did not include data from Japan or China. However, our prevalence results are consistent with those reported in recent studies from Japan;^39^,^40^ (range, 11%-27%) and China^41^(20%). Finally, any literature survey is retrospective in nature and, as we observed, is subject to data heterogeneity. Among other factors, referral bias may have affected the prevalence reported by some researchers, particularly those from specialist centers. Whether the data in our selected studies are representative of the wider population of breast cancer patients in these countries cannot be determined until further studies are conducted. Nevertheless, as regional breast cancer experts, we believe the available data, although limited, are consistent with our clinical experience.

In summary, data on the prevalence of ErbB2-positive breast cancer in Asia are limited. Nevertheless, our survey, based on data from 14,398 patients in 7 Asian countries, indicates that the reported prevalence of ErbB2-positive breast cancer has varied widely from 6% to 65%. However, when ErbB2 status was assessed robustly, the median prevalence, based on data from 10,076 patients in 5 Asian countries, was 19% (IHC3+) or 25% (FISH), similar to that in the West. Because ErbB2 status is predictive of both prognosis and responsiveness to targeted therapies, Asian countries should work toward standardization of ErbB2 assessment.

## CONFLICT OF INTEREST DISCLOSURES

This study was sponsored by Glaxo SmithKline Asia Pacific (Singapore). Dr. Aggarwal has acted as a consultant for Glaxo SmithKline, AstraZeneca, Dabur, and Roche, and has received honoraria from GlaxoSmithKline, Bristol-Myers Squibb, AstraZeneca, and Merck. Drs. Yip and Sunpaweravong have acted as consultants for GlaxoSmithKline. Drs. Yeoh and Moon are employees of GlaxoSmithKline Asia Pacific. The authors acknowledge the independent medical writing assistance provided by ProScribe Medical Communications (http://www.proscribe.com.au [accessed July 23, 2010]), funded from an unrestricted financial grant from GlaxoSmithKline Asia Pacific. ProScribe's services complied with international guidelines for Good Publication Practice.
